# Adrenal Crest in Testicular Tissue of an Undescended Testis in an Adult Patient: A Case Report

**DOI:** 10.7759/cureus.47640

**Published:** 2023-10-25

**Authors:** Mohand A Alzughaibi, Abeer Alasiry, Khadijah Eid, Mohammed Alshehri, Reham A Khubrani

**Affiliations:** 1 Urology, King Abdullah bin Abdulaziz University Hospital, Riyadh, SAU; 2 Medicine and Surgery, Princess Nourah Bint Abdulrahman University, Riyadh, SAU; 3 Basic Sciences, Princess Nourah Bint Abdulrahman University, Riyadh, SAU; 4 Pathology and Laboratory Medicine, King Abdullah bin Abdulaziz University Hospital, Riyadh, SAU

**Keywords:** adult population, ectopic adrenal tissue in spermatic cord, eat and udt, undescended testis, heterotopic or ectopic adrenal cortical tissue (eact)

## Abstract

This case report describes a rare occurrence of ectopic adrenal cortical tissue (EACT) in the undescended testis (UDT) of an adult male patient. The patient presented with an empty left scrotum since birth, and a magnetic resonance imaging (MRI) confirmed the diagnosis of left-side UDT. Orchidectomy was performed, and a microscopic examination revealed a UDT with EACT. The patient had no significant medical or surgical history and had a normal preoperative hormonal profile. EACT is usually found incidentally during surgical procedures and is more common in children than adults. This case emphasizes the importance of investigating adrenal rests due to the potential of neoplastic transformation or hormonal activity. The case report concludes that EACT of the UDT is still a possibility in the adult patient population.

## Introduction

Discovering heterotopic adrenal tissue along the spermatic cord is a rare medical condition. During embryological development, many structures would migrate from their initial position in the vicinity of the adrenal gland to their final position in the abdomen or the pelvis. Such structures can harbor heterotopic adrenal glandular tissue. Retroperitoneal adipose tissue and the kidneys are the usual recipients of ectopic adrenal tissue. In comparison, the ovary, testis, spermatic cord, liver, colon transversum, and gastric wall are rarely reported with adrenal heterotopia [1].

Our case presented with an empty left scrotum since birth. On clinical examination, the testis was absent in the left scrotal sac, which was confirmed with magnetic resonance imaging (MRI). The clinical diagnosis was a left-side undescended testis (UDT). Orchidectomy was conducted, and on microscopic examination, it was consistent with a UDT with ectopic adrenal cortical tissue (EACT). We present this case that illustrates the unusual and rare occurrence at this age and site.

## Case presentation

**Figure 1:**
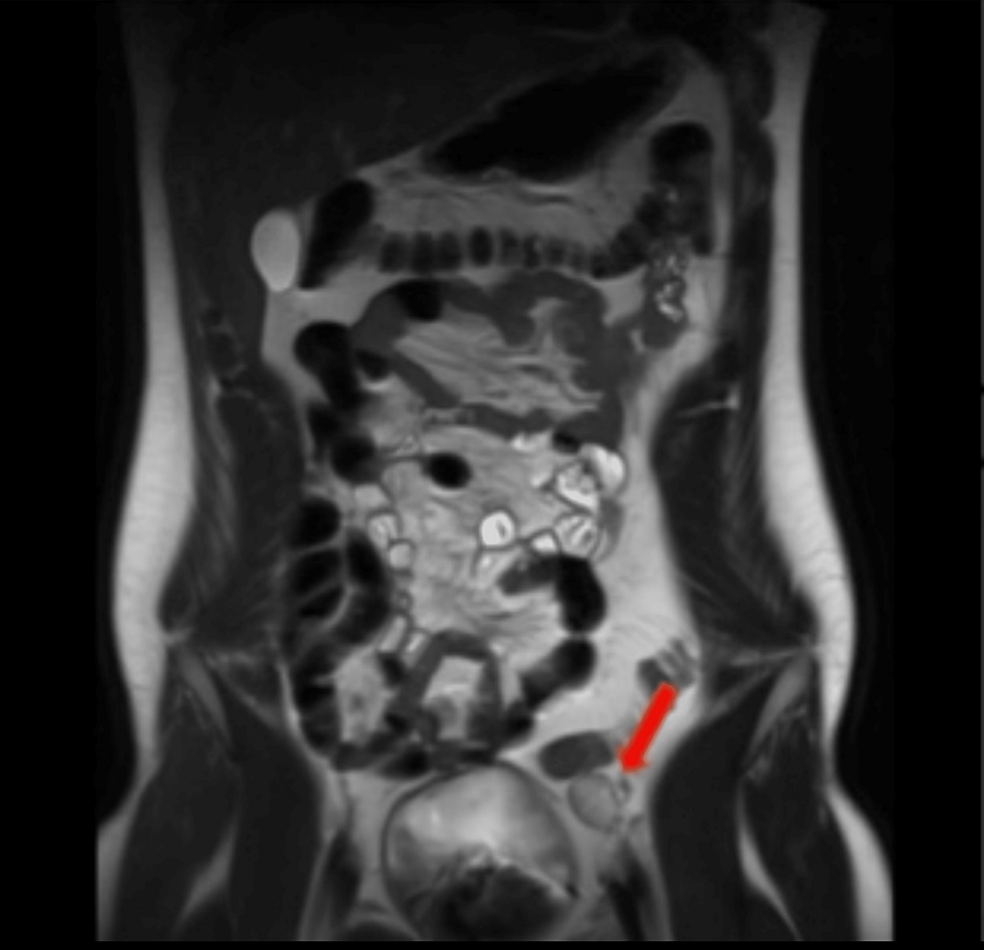
A multi-sequential, multi-planer abdominal pelvic MRI (coronal view) that was performed in November 2022 with IV contrast administration. Findings were consistent with a well-defined, 2.0 cm x 1.7 cm x 2.2 cm oval homogenous T2 intermediate to hyperintense structure. The testis was located approximately 2 cm proximal to the deep inguinal ring within the pelvic cavity. MRI, magnetic resonance imaging; IV, intravenous

**Figure 2:**
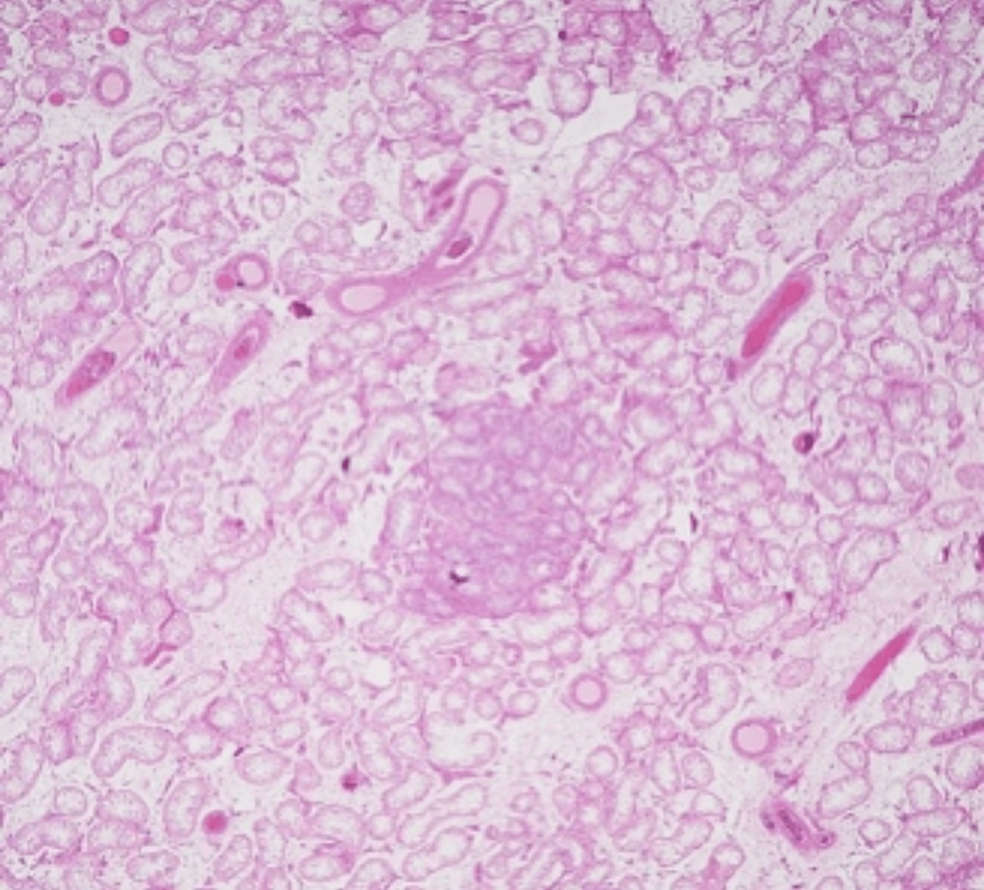
The hematoxylin-and-eosin-stained section from the undescended testicle shows atrophic seminiferous tubules and Sertoli cell adenoma.

**Figure 3:**
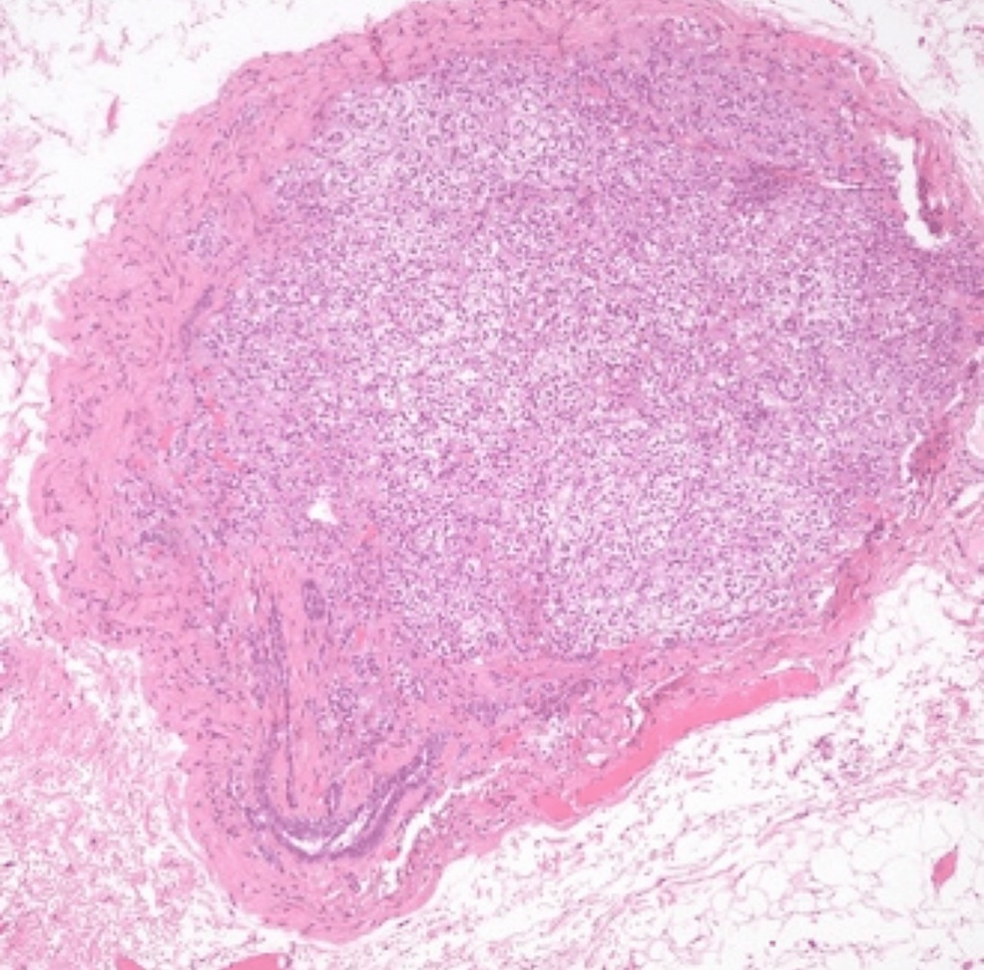
The hematoxylin-and-eosin-stained section shows a nodule of mature adrenal gland tissue.

A 21-year-old male, with no significant medical or surgical history, presented to our clinic with an empty left scrotum since birth. The patient reported noticing a 2 cm x 2 cm bulge in his left inguinal area that can sometimes be palpated. Upon examination, the left hemiscrotum was noted to be atrophic, exhibiting minimal rugae in comparison to the opposite side. The left inguinal region was smooth with no obvious lump, which can be consistent with a UDT. The right testis was hypertrophic with normal epididymis and palpable vas but no varicocele. A preoperative hormonal profile was conducted to rule out testicular malignancy. Lactate dehydrogenase, human chorionic gonadotropin, and alpha-fetoprotein were all within normal limits. To confirm the testicular position, an MRI was ordered (Figure 1). Findings were consistent with a well-defined, 2.0 cm x 1.7 cm x 2.2 cm oval homogenous T2 intermediate to hyperintense structure. The testis was within 2 cm proximal to the deep inguinal ring within the pelvic cavity. The patient underwent laparoscopic exploration and orchiectomy. Intraoperatively, no Mullerian remnants could be identified. The gross pathology report showed an orchidectomy specimen measuring 2.5 cm x 2.0 cm x 1.5 cm and the epididymis measuring 3.5 cm x 0.5 cm x 0.5 cm. The outer testicular surface was smooth and shiny. The cut surface was white, tan, and solid. No focal lesion was seen. Microscopically, the specimen was consistent with a UDT with ectopic adrenal cortical tissue. The specimen was negative for malignancy (Figures 2-3).

## Discussion

UDT is a prevalent congenital anomaly observed among pediatric patients. Anomalies of the epididymis have been documented in connection with undescended testicles. While it's acknowledged that the adrenal cortex can sometimes develop in atypical locations, instances of adrenal tissue developing outside its usual location near the adrenal gland are infrequent. Only a handful of cases of ectopic adrenal tissue in the para-testicular and inguinal regions have been reported, and this occurrence is even rarer in adults [2].

Heterotopic adrenal cortical tissue or EACT can be located in the upper abdominal region or along the trajectory of the gonads' descent. The connection between the developing adrenal cortex and the genital ridge during embryonic development clarifies the correlation with the gonads. Ectopic adrenal tissue is believed to arise when cells detach from the primary body of the adrenal cortex or emerge outside of it, subsequently attaching to or becoming associated with the gonads or nearby tissues [3]. Following this, the ectopic tissue has the potential to migrate to distant sites alongside the gonad. The adrenal medulla, on the other hand, forms independently as cells from the sympathetic ganglia migrate and then infiltrate the adrenal cortex around its central vein. This phenomenon could elucidate why ectopic adrenal nodules situated farther from the adrenal gland usually consist exclusively of the adrenal cortex [3].

The discovery of ectopic adrenal tissue within the spermatic cord has primarily occurred as an unintended finding during surgical interventions, such as herniotomy or orchiopexy, in the inguinoscrotal region [4]. These rests are usually asymptomatic. Rarely, they can grow to be larger and attract clinical attention. Similarly, this ectopic adrenal tissue can undergo a neoplastic transformation or become functional, releasing hormones, and causing various symptoms resulting in significant morbidity and mortality [5]. In the early 1900s, a case of a functional tumor arising in an adrenal cortical rest located in the broad ligament was reported. Several such neoplasms have been identified since [6].

These heterotopic rests are more common in children than in adults. In their study of 13 cases, Mendez et al. discovered that the median age of diagnosis was 5.8 years. They found that ectopic rests are ten times more common in children than in adults. However, in our case, the patient was an adult male who presented with a UDT [7].

## Conclusions

To summarize, EACT represents a rare form of pathology, often only detected accidentally during surgical interventions. There is, however, a notable surge in its occurrence in conjunction with UDT. This escalation may be attributed to the embryological activities involved in the development of the adrenal and gonadal systems. The correlation between EACT and UDT might also provide insights into theories concerning testicular descent, particularly focusing on the formation of the gonadal ridge and the migration of primordial germ cells.

In a surgical approach to a patient, further investigations are critical of these adrenal rests due to the possibility of these ectopic adrenal tissues undergoing neoplastic transformation or becoming functional. The significance of our case is that despite its rare occurrence, the EACT of the UDT is still a possibility in the adult patient population.
